# Sexual violence among LGB+ South Asian Americans: Findings from a community survey

**DOI:** 10.1371/journal.pone.0264061

**Published:** 2022-02-24

**Authors:** Shahmir H. Ali, Sadia Mohaimin, Ritu Dhar, Moitrayee Dhar, Farzana Rahman, Liza Roychowdhury, Tanzeela Islam, Sahnah Lim

**Affiliations:** 1 Department of Social and Behavioral Sciences, School of Global Public Health, New York University, New York, NY, United States of America; 2 Department of Population Health, Grossman School of Medicine, New York University, New York, NY, United States of America; Universita degli Studi della Campania Luigi Vanvitelli, ITALY

## Abstract

Lesbian, gay, bisexual, and other sexual minority (LGB+) South Asian Americans represent a disproportionately underserved and often invisible community in the United States. While issues of sexual violence have been documented in the South Asian American community, little is known on its impact among LGB+ individuals. This study explores the experience of sexual violence, related attitudes, and mental health outcomes among LGB+ South Asian Americans. A community-informed online survey of 18–34-year-old South Asian Americans living near the New York State region, recruited from online social media platforms, was conducted. Study design, implementation, and evaluation occurred in partnership with an advisory board of South Asian young adult representatives; data was analyzed both descriptively and through multivariable logistic regression models. Of the 385 participants who reported their sexuality, LGB+ participants comprised 24.1% (n = 93) of the sample. LGB+ participants were more likely to have experienced rape multiple times (17.2% vs. 9.6%) in bivariate analyses, and higher odds of depression (AOR:3.47, 95%CI:1.61–8.17) in adjusted analyses. Overall, LGB+ South Asian Americans displayed a disproportionate burden of sexual violence and depression. Findings identify policy and research pathways to address sexual violence among LGB+ South Asians.

## Introduction

South Asian Americans (i.e. individuals who trace their ethnic heritage from the countries of India, Pakistan, Bangladesh, Sri Lanka, and Nepal) are now the fastest growing racial or ethnic minority group in the United States (US) [1,2]. This diverse community experiences a complex health burden involving both physical health (e.g. diabetes and heart disease) and mental health (e.g. depression) concerns [3,4]. Contributing to this health burden experienced by many South Asian Americans are structural obstacles related to socio-economic disadvantage, linguistic barriers to service utilization, as well as social and cultural factors which underpin a unique landscape of health-related knowledge, attitudes, and behaviors [5,6]. However, there has been growing attention to the prevalence of sexual violence among South Asians, with preliminary evidence suggesting that between 21.2% to 40.8% of South Asian American women reporting some form of intimate partner violence [7], which has also been associated with higher reports of depression and anxiety [8]. Sexual violence and its associated health impact among South Asian Americans has been linked with culturally and religiously driven stigma, lack of community-based prevention initiatives, and inaccessibility of services for survivors [9].

However, disaggregating the experience and impact of sexual violence within the South Asian American community is vital to appropriately identify points of intervention. For example, little is known of the health experiences of sexual minorities in the South Asian community. Faced with both intra- and inter-community social forces contributing to an intersectional experience of marginalization, South Asian immigrants identifying as lesbian, gay, bisexual, or otherwise not heterosexual (LGB+) have been shown to experience additive and interactive effects of minority stress driven by discrimination, racism, internalized heterosexism, acculturation, and enculturation [10]. However, LGB+ South Asians have remained a largely invisible population in the US [11], in part due to disclosure stigma associated with cultural and religious values of heteronormativity and its association with familial expectations, honor, and pride [12]. Importantly, in the context of sexual violence, this preliminary sociological evidence suggests that LGB+ South Asian Americans and other sexual and gender minority Asian Americans may experience a disproportionate health impact due to traumas related to interpersonal and structural discrimination, differences in the type of social support accessible to survivors, and the confluence of stigmas related to both sexual assault disclosure and one’s sexuality [13]. Given that LGB+ Americans as a larger community already experience obstacles in reporting sexual violence or receiving services (e.g. discrimination in hospital settings) [14], for LGB+ South Asian Americans, these same barriers may further intersect with socio-economic status, immigration, or other cultural and religious obstacles experienced by South Asians at large. The aim of this study is to describe and disaggregate the experiences of sexual violence among LGB+ South Asian Americans and evaluate differences in sexual violence related attitudes and mental health outcomes between LGB+ and heterosexual South Asian Americans.

## Methods

Informed by principles of community-based participatory research (CBPR), an advisory board of South Asian young adult representatives in NYC was created to inform the study’s development, implementation, evaluation, and dissemination. The board was formed following a wave of disclosures of sexual assault on social media by young South Asian women in early 2020. Self-identifying 18–34-year-old South Asian residents of the NYC area (including those residing or staying in New York, New Jersey, and Connecticut) were recruited via social media (notably posts made on Instagram and Facebook) to participated in an online, self-administered survey on sexual assault and mental health related outcomes. Social media has been identified as a valid and effective method to recruit geographically dispersed, hard-to-reach populations, notably during the COVID-19 pandemic (when data collection occurred) [15,16]. Recruitment posts were particularly disseminated to the social media pages of organizations likely catering to South Asian young adult populations (e.g., student associations and South Asian advocacy groups), which assisted in the iterative sharing of the study information to maximize reach. Along with socio-demographic background, participants were asked to identify their sexual orientation informed by categories used in past surveys [17]: straight, lesbian or gay, bisexual, or other. Participants were instructed to read through elements of the study and then consent was implied via “clicking next” to the first question of the survey. All study materials were approved by the New York University Grossman School of Medicine Institutional Review Board.

Experience of sexual violence was assessed using adapted items from the Sexual Experiences Survey Long Form Victimization (SES-LFV) [18]. Questions on sexual assault attitudes were adapted from past scales [19–21] and consultations with the partnering advisory board. Although most attitude items were analyzed individually, exploratory factor analysis identified a 5-point score of attitudes towards bystander behaviors constructed of two items on acceptability (strongly agree to disagree) towards participating in activities where 1) a person’s attractiveness is ranked, 2) nude photos/videos are shared. Moderate to severe depression was assessed with a score of 15 or higher on the 10-item Centre for Epidemiologic Studies Depression Scale (CESD-10) [22], and post-traumatic stress disorder (PTSD) through the PTSD Checklist for Diagnostic and Statistical Manual of Mental Disorders (PCL-5) [23]. At the end of the survey, a list of culturally tailored local resources related to sexual violence were also provided.

Bivariate analyses of differences in mental health and sexual assault outcomes by sexual minority status (heterosexual or LGB+) were conducted using chi-square tests for significance (p<0.05), which informed multivariable logistic regression analyses of these outcomes. Models were adjusted by age, sex, US-born status, and religion; the primary sexual violence and mental health variables analyzed were lifetime experience of multiple rape (>3 times) and moderate to severe depression. Additional analyses of lifetime experience of rape and PTSD were also conducted. Analyses were conducted on R (version 4.0.2).

## Results

A total of 385 responses were recorded with data on sexual identity (98.0% of all recorded responses); LGB+ participants comprised 24.1% (n = 93) of the sample (Table 1). Most participants reported experience some form of sexual violence (85.5%), including both no-contact-based (82.6%) and contact-based (62.9%) sexual violence. Prevalence of rape (37.1%) and multiple rape (11.4%) experiences were notably high, along with symptoms of depression (60.3%) and PTSD (47.8%).

**Table 1:** Characteristics and significant sexual assault related disparities among sample of South Asian young adults stratified by sexual minority status (N = 385).

		Total (n = 385)		Heterosexual (n = 292)		LGB+ (n = 93)		p-value	UOR	AOR
		N/mean	%/SD	N/mean	%/SD	N/mean	%/SD			
Age, mean (SD)		23.1	3.6	23.2	3.8	22.7	3.5	0.258	0.96 (0.90–1.03)	0.91 (0.82–1.00)
Sex at birth	Male	61	15.8%	49	16.8%	12	12.9%	0.513	0.75 (0.37–1.45)	0.91 (0.34–2.30)
Religion	Muslim	194	50.4%	158	54.1%	36	38.7%	0.013	*0.53 (0.33–0.86)	0.61 (0.26–1.50)
	Hindu	72	18.7%	56	19.2%	16	17.2%	0.786	0.88 (0.46–1.59)	0.72 (0.24–2.14)
	Ath./Agn./Not-Relig.	41	10.6%	24	8.2%	17	18.3%	0.011	**2.50 (1.26–4.88)	1.31 (0.42–4.09)
US-born	Yes	283	73.5%	211	72.3%	72	77.4%	0.397	1.32 (0.77–2.32)	0.90 (0.43–1.94)
Sexual violence	Any kind	329	85.5%	242	82.9%	87	93.5%	0.074	2.40 (1.05–6.57)	-
	No contact	318	82.6%	233	79.8%	85	91.4%	0.078	2.14 (1.02–5.07)	-
	Contact	242	62.9%	174	59.6%	58	62.4%	0.041	*1.17 (1.06–3.04)	-
	Rape Attempt	162	42.1%	117	40.1%	45	48.4%	0.145	1.47 (0.91–2.37)	-
	Rape	143	37.1%	102	34.9%	41	44.1%	0.094	1.56 (0.96–2.53)	-
	Multiple Rape (>3 times)	44	11.4%	28	9.6%	16	17.2%	0.037	*2.21 (1.09–4.39)	2.06 (0.91–4.61)
Attitudes	Attitudes towards bystander participation	1.29	0.47	1.27	0.43	1.37	0.56	0.072	1.53 (0.95–2.43)	1.69 (0.87–3.36)
	Victim can continue relationship with perpetrator	173	44.9%	125	42.8%	48	51.6%	0.075	1.60 (0.99–2.59)	2.01 (0.68–5.76)
	Victim can begin relationship with perpetrator	150	39.0%	107	36.6%	43	46.2%	0.079	1.58 (0.98–2.56)	1.08 (0.39–3.16)
Mental health	Depressive symptoms	232	60.3%	163	55.8%	69	74.2%	0.001	**2.82 (1.57–5.37)	**3.47 (1.61–8.17)
	Post-traumatic stress disorder	184	47.8%	127	43.5%	57	61.3%	0.042	**1.73 (1.05–2.86)	-

*p<0.05

**p<0.01

***p<0.001.

In bivariate analyses, LGB+ participants were more likely to have experienced contact-based sexual violence (62.4% vs. 59.6%) and rape multiple times (17.2% vs. 9.6%) compared to their heterosexual counterparts. However, in adjusted analyses, odds of multiple rape did not statistically differ by sexual minority status when adjusted for either depression or PTSD, nor did odds of rape (not shown). LGB+ participants reported a higher prevalence of both moderate to severe depression (74.2% vs. 55.8%) and PTSD (61.3% vs. 43.5%), including higher odds of depression in adjusted analyses (AOR:3.47, 95%CI:1.61–8.17) when adjusted for either rape or multiple rape; however, odds of PTSD were not significant in either of the adjusted analyses (not shown).

## Discussion

Overall, differences and specific attitudes and outcomes related to sexual violence were observed between LGB+ and heterosexual South Asian young adults. Given the significant lack of research and invisibility of LGB+ South Asian young adults [11], this study provides crucial preliminary insights into the types of sexual violence among this underserved population. The proportion of LGB+ participants in the sample (24.1%) was notably high and, although similar to the portion of LGB+ participants in past surveys of young South Asian Americans [10], may also be a reflection of the recruitment methods (which relied upon networks of South Asians active on social media, including many advocacy pages which may cater to LGB+ communities). Although the prevalence of sexual violence in the total sample was observed to be higher than other preliminary estimates among South Asian Americans [7], bivariate analyses suggested that LGB+ South Asian young adults were more likely to experience multiple rape and contact-based sexual violence, with other forms of violence also approaching statistical significance. Thus, policy action to reduce sexual violence among South Asian Americans must also target the violence burden experienced by the LGB+ community, and findings call for further scaled-up research to better understand factors behind why LGB+ South Asian Americans may experience certain forms of sexual violence disproportionately.

Although significant differences in sexual violence related attitudes were not observed (likely due by sample size constraints), among those that were examined, positive attitudes towards the ranking of physical attractiveness and sharing of nude photos among LGB+ South Asians particularly approached significance in bivariable analyses (p = 0.072). There has been growing evidence to suggest the importance of body image and perceived physical attractiveness among LGB+ young adults, particularly the role of media and community standards in catalyzing physical standards of belongingness withing LGB+ sub-communities [24]. Acceptability of ranking physical attractiveness among LGB+ South Asians may be an indicator of this importance of physicality observed across the broader LGB+ community, and may represent a point of intervention among South Asians to help curb underlying issues of body image stigma. Furthermore, acceptability of sharing nude photos may also reflect differences in the culture of popular LGB+ dating applications, such as Grindr, to share nude photos and other aspects of LGB+ "hook-up" culture prioritizing sexual over romantic interactions [25]. The prevalence of sexual image sharing may also highlight why LGB+ internet users are more likely to have experienced threats of (or actual) non-consensual image-sharing [26], thus study findings call for additional research to better understand this phenomena and potentially for promotional efforts towards LGB+ South Asians aimed at reducing risky or non-consensual practices of sexual image sharing.

The proportion of participants reporting symptoms of depression and PTSD, both LGB+ (74.2%, 61.3%) and heterosexual (55.8%, 43.5%), were significantly higher than the estimates of the US average prevalence of depression (8.1%) [27] and 1-year prevalence of PTSD (2.3–9.1%) [28], which may suggest a significantly burden of mental health challenges among South Asian young adults (particularly those identifying as LGB+). Higher odds of depression among South Asian LGB+ young adults also support past evidence highlighting elevated symptoms of depression among sexuality minority youth compared to heterosexual youth [29]. Indeed, limited utilization of psychological services has been an issue identified in diverse LGB+ communities [30], with intra-community and inter-community discrimination particularly significant barriers to health-care utilization for LGB+ South Asian immigrants [31]. While interventions targeting depression have been limited across the LGB+ community, the unique social, cultural, and interpersonal stressors faced by South Asian LGB+ young adults may require a tailored approach to mental health interventions [12,13]; further research on mental healthcare utilization and contributors to the expression of depression among LGB+ South Asians is warranted.

## Conclusion

Within the already underserved population of South Asian Americans, LGB+ young adults represent a subgroup whose complex health needs remain unknown. By providing crucial first insights into attitudes and experiences related to sexual violence among LGB+ South Asian Americans, our findings identify pathways for intervention, including the potentially disproportionate rape and contact-based sexual assault burden, as well as attitudes related the physical appearance and the dissemination of sexual images. Importantly, given the preliminary, formative nature of the available data analyzed (which involved convenience sampling with limitations in systematically assessing response rates), strong conclusions on the complex health and social experiences of LGB+ South Asians cannot be inferred. Building on these findings, further quantitative and qualitative research is warranted to better understand underlying contributors and mechanisms behind the health experiences, behaviors, and attitudes among LGB+ South Asians (including disparities across socio-economic, geographic, and ethnicity outcomes not assessed in the current survey) to inform improved health policy.
